# Case Report: A Review of the Literature on Spinal Intradural Hemangiopericytoma With Spinal Cord Infiltration and a Case Report

**DOI:** 10.3389/fsurg.2020.600563

**Published:** 2020-12-16

**Authors:** Liu Chunyang, Zhu Huiqin, Sun Mo, Wang Yubo, Zhang Xianfeng

**Affiliations:** ^1^Department of Neurosurgery, First Hospital of Jilin University, Jilin, China; ^2^Department of Respiratory Medicine, First Hospital of Jilin University, Jilin, China; ^3^Department of Pathology, First Hospital of Jilin University, Jilin, China

**Keywords:** hemangiopericytoma, spinal tumor, intraspinal canal, intramedullary, treatment

## Abstract

**Purpose:** Primary spinal intradural hemangiopericytoma (HPC) with spinal cord infiltration is rare. The purposes of this study were to investigate the clinical features of intradural HPC with spinal cord infiltration and to explore the related factors affecting tumor recurrence.

**Methods:** We report a case of intramedullary HPC with intramedullary infiltration of the thoracic spine. The relevant literature was searched for with PubMed, and clinical data were extracted from the included studies. Clinical patient data were described and statistically analyzed. Then, Kaplan-Meier (KM) curves were used to describe the relapse-free survival (RFS) of patients in different groups, and the log-rank test was used for evaluation.

**Results:** A total of 11 cases of spinal intradural HPC with spinal cord infiltration were included (including the case described in this report). Further data analysis showed that sex (*P* = 0.249), age (*P* = 0.876), tumor location (*P* = 0.524), and postoperative radiotherapy (*P* = 0.12) had no significant influence on RFS. The range of tumor resection (*P* = 0.004) and the WHO grade (*P* = 0.014) significantly affect the patient RFS.

**Conclusion:** RFS was higher in patients with total tumor resection than in patients with subtotal tumor resection. The patients with lower WHO grade have better RFS. Total tumor resection is the primary objective of surgical treatment of spinal intradural HPC with spinal infiltration. Long-term postoperative follow-up is considered necessary.

## Introduction

Hemangiopericytoma (HPC) is a rare vascular tumor originating from Zimmermann peripheral cells, which were first described by Stott and Murray (1). Characterized by a highly vascular and invasive nature, HPC has a strong tendency for local recurrence and metastasis. The local recurrence rate and metastasis rate of HPC are 60 and 23%, respectively (2). Primary spinal intradural HPC is rare. We reported a rare case of intradural HPC of the thoracic vertebra (T4-5) with spinal cord infiltration and searched the relevant literature for statistically significant data.

## Methods

We report a male patient with thoracic MRI showing a horizontal intradural space-occupying lesion of the T4-5 vertebral bodies. The tumor was removed by posterior laminectomy and found to infiltrate the dorsal spinal cord. Microscopically, the tumor was resected as a whole, and histopathology and immunohistochemistry suggested HPC (WHO grade II). A PubMed search for cases and series related to spinal HPC published as of May 20, 2020 was conducted with “Neurosurgical diseases,” “intraspinal” and “hemangiopericytoma” used as keywords. In addition, references cited in the selected articles were manually searched and reviewed to identify other potentially eligible studies. The patient data were extracted, and the clinical patient data were described and counted. Then, Kaplan-Meier (KM) curves were used to describe the relapse-free survival (RFS) of the patients in different groups, and the results were evaluated by log-rank test. The inclusion criteria were as follows: (1) tumor histopathologically diagnosed as HPC located in the spinal intradural space, (2) intraoperative tumor infiltration of the spinal cord, (3) no available overlapping information, (4) adequate follow-up time (at least 6 months), and (5) study published in English. Only cases of spinal intradural HPC with spinal cord infiltration were analyzed, excluding cases of spinal epidural HPC and spinal intradural HPC without spinal cord infiltration. The whole screening process was independently completed by two authors (Liu Chunyang and Zhu Huiqin) according to the inclusion and exclusion criteria. The corresponding authors (Yubo Wang and Xianfeng Zhang) should be consulted for any disagreements.

## Clinical Case

**Figure 1:**
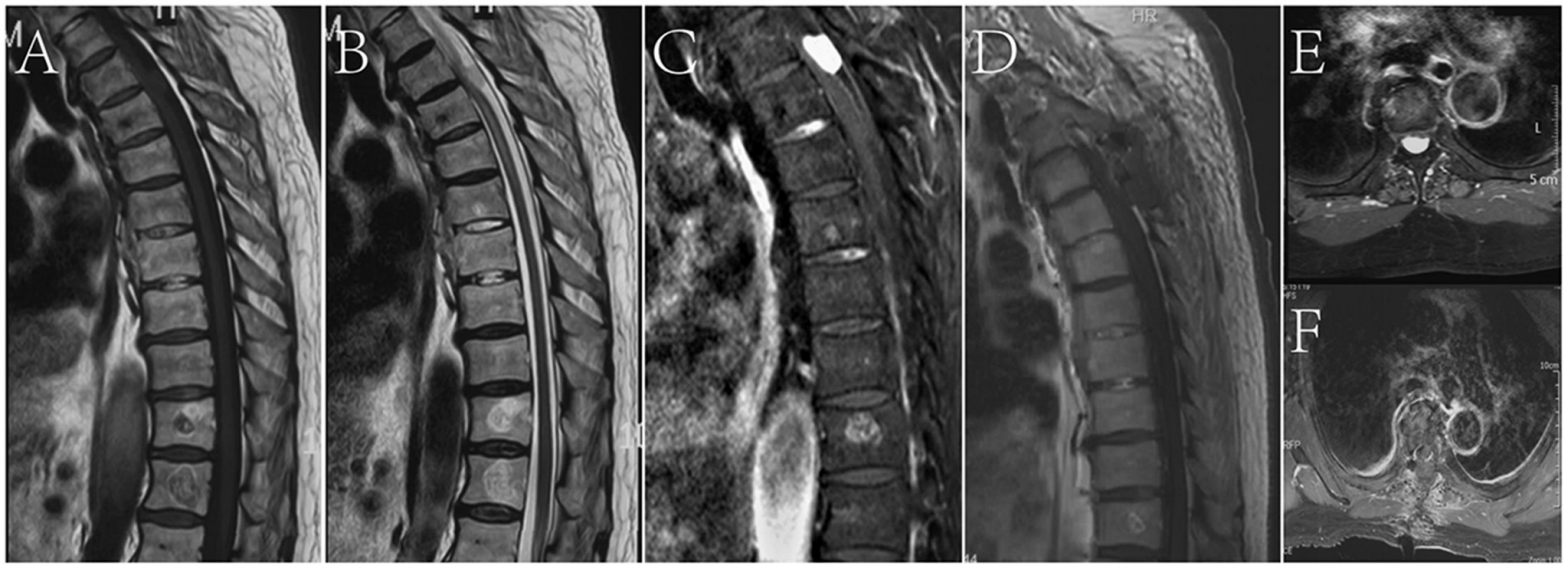
**(A,B)** The tumor presented isosignals on T1WI and T2WI. **(C,E)** The mass showed uniform enhancement after contrast agent injection. **(D,F)** Post-operative review by thoracic MRI showed that all tumors had been removed.

An 80-year-old male patient with weakness of both lower extremities was admitted to the hospital for more than 1 month. The patient described progressive incomplete paralysis of both lower extremities. Thoracic MRI showed a horizontal intradural space-occupying lesion of the T4-5 vertebral bodies. T1WI and T2WI presented isosignals (Figures 1A,B) and were uniformly enhanced after gadolinium-diethylenetriaminepentaacetic acid (GD-DTPA) injection (Figures 1C,E). The tumor was removed by T4-5 laminectomy under neurophysiological examination. During the operation, the tumor was found to be dark red with a vascularized surface and eroded dorsal spinal cord and nerve roots. The interface of the tumor tissue where it infiltrates the spinal cord was carefully separated under a microscope, and the whole tumor was removed (Figures 1D,F) with minimal bleeding. The lower limbs could move freely after the operation. After 10 months of follow-up, the patient died of heart disease.

**Figure 2:**
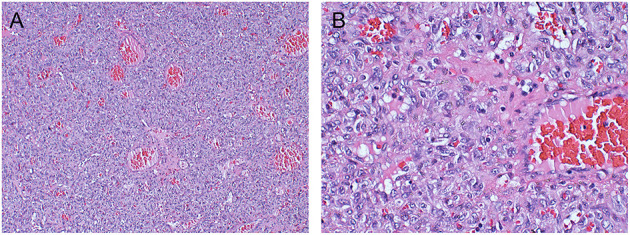
**(A)** At low magnification, the tumor tissue is rich in cells showing a rounded oval to spindle-like shape, and the intercellular stroma is rich in “antler-like” parenchyma branching vessels (HE × 100). **(B)** Mitotic image is not easy to be seen in high magnification (HE × 400 mm).

**Figure 3:**
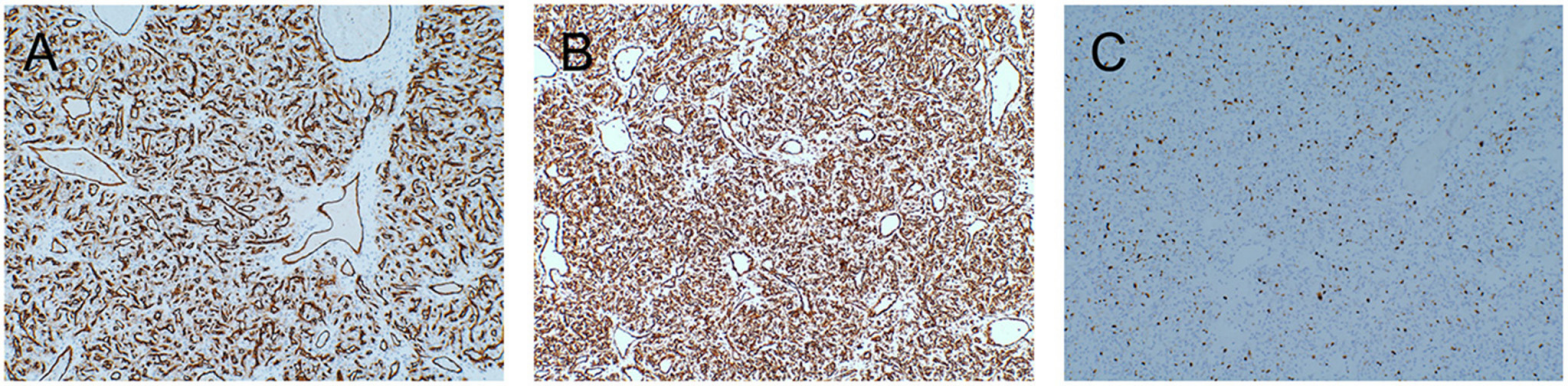
Both CD34 **(A)** and Vimentin **(B)** were positive by immunohistochemical staining. The Ki-67 index was ~20% **(C)**.

Postoperative histopathology showed WHO grade II HPC. Under light microscopy, the tumor tissue was rich in cells showing a rounded oval to spindle-like shape, and the intercellular stroma was rich in “staghorn” thin-walled branching vessels (Figure 2A). Mitotic figures were not easy to see (Figure 2B). Immunohistochemical staining was Ki-67(+20%), CD34(+), Vimentin(+), SSTR2(–), EMA(–), STAT6(–), D2-40(–), and GFAP(–) (Figure 3). These results are consistent with the characteristics of HPC.

## Results

A total of 33 studies from 1965 to 2020 consisting of 112 cases of HPC of the spine were found by a Medline search. Among the cases, only 10 cases were spinal intradural HPC with spinal cord infiltration (Table 1) (3–6). We performed statistical analysis of the data in the literature with the statistical software package SPSS, and the data are expressed as the mean ± standard deviation. All the data were analyzed by the software of IBM SPSS Statistics 26 (IBM Corporation, Armonk, New York, USA). Due to the limited number of cases, we performed univariate analysis of the data to determine factors associated with patient survival. Clinicopathologic variables included sex, age, tumor location, surgical scope, and postoperative radiotherapy. After statistical treatment, the difference was statistically significant (*P* < 0.05).

**Table 1:** Summary of the literature on spinal epidural hemangiopericytoma with spinal cord infiltration.

**Reference**	**Age/sex**	**Starting symptoms**	**Duration of Symptoms**	**Tumor location**	**Surgical treatment**	**Postoperative radiotherapy**	**Recurrence**	**Transfer**	**Follow-up time**	**WHO grade**	**Survival**
Wang et al. (3)	38/M	Back pain	25 m	T5-7	STR	No	Yes	Lung	26 m	III	Dead
	66/M	Back pain; lower limb weakness	14 m	L2-3	STR	No	Yes		25 m	III	Dead
	47/F	Back pain; lower limb weakness	10 m	L1	GTR	Yes	No		82 m	II	Alive
	48/M	Back pain; lower limb weakness	6 m	T9-10	STR	No	Yes		21 m	III	Alive
	42/F	Neck pain	4 m	C5-6	GTR	Yes	No		76 m	II	Alive
	46/F	Defecation and defecation disorder	6 m	L3-5	STR	No	Yes	Lung	19 m	III	Dead
Liu et al. (4)	32/F	NR	NR	T5-6	GTR	Yes	No		80.4 m	II	Alive
	24/M	NR	NR	C5-7	GTR	Yes	No		21.6 m	II	Alive
Chou et al. (5)	80/M	Weakness of both lower limbs and urinary incontinence	3 m	T10	GTR	No	No		36 m	NR	Alive
Das et al. (6)	34/M	Progressive paralysis	6 m	T8-10	STR	Radiotherapy and chemotherapy	Yes	Multiple metastases	24 m	III	Dead
Present case	80/M	Progressive weakness of both lower limbs	1 m	T4-5	GTR	No	No		8 m	II	Alive

*F, female; M, male; GTR, gross total resection; STR, subtotal resection; NR, not reported; m, month*.

**Figure 4:**
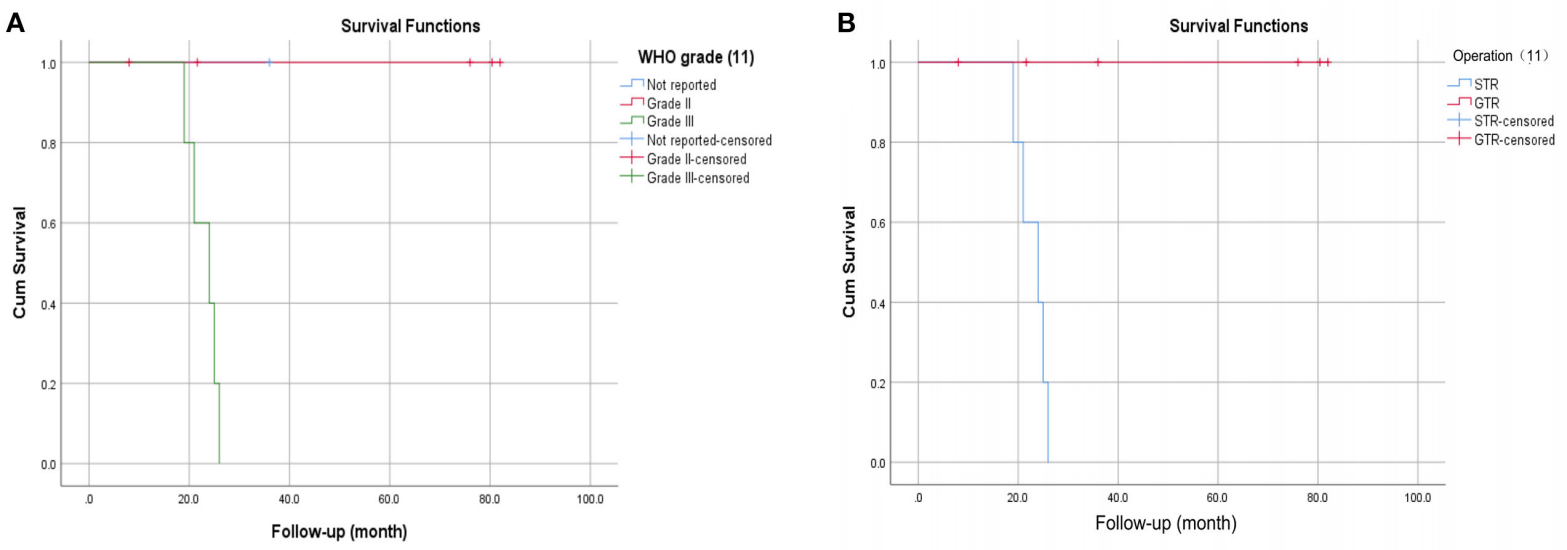
Kaplan-Meier survival curves showing the WHO grade **(A)** and tumor resection range **(B)** significantly affected the RFS.

Table 1 summarizes the clinical data of 11 patients with spinal intradural HPC with spinal cord infiltration. Among them were seven males and four females, with a mean age of 48.82 ± 18.80 years (median age was 46 years old, ranging from 24 to 80 years old). Weakness of the lower extremities was the most common first clinical symptom and often followed a progressive process. Other symptoms included neck and back pain and trouble urinating. The tumors were located in the thoracic spinal cord in six cases (54.5%), cervical spinal cord in two cases (18.2%), and lumbar spinal cord in three cases (27.3%). All patients underwent MRI scanning; T1WI showed low signal or isosignal, T2WI showed high signal (100%), and enhanced images showed uniform or uneven enhancement. All patients received surgical treatment, including five patients (45.5%) treated with subtotal tumor resection and six patients (54.5%) treated with total tumor resection. Postoperative radiotherapy was carried out in five patients (including chemotherapy in one patient). All patients were followed up for more than 6 months, with an average follow-up time of 38.1 ± 27.4 months (median 24.5 months, range 8–82 months). The recurrence rate was 45.5% (five patients, all of whom underwent subtotal tumor resection). The mean time of recurrence was 23 ± 2.92 months (median 24 months, range 19–26 months). Among the five patients with recurrence, all had WHO grade III HPC, 4 patients did not receive adjuvant therapy, and 1 patient received postoperative radiotherapy and chemotherapy. Distant metastases occurred in three patients, the most common site of metastasis being the lung, giving a metastasis rate of 27.3%. Further analysis of the data showed that sex (*P* = 0.249), age (*P* = 0.876), tumor location (*P* = 0.524), and postoperative radiotherapy (*P* = 0.12) had no statistically significant impact on the RFS. The WHO grade (*P* = 0.014) (Figure 4A) and the scope of tumor resection (*P* = 0.004) (Figure 4B) significantly affected the RFS rate of patients.

## Discussion

In 2016, the WHO used the combined term solitary fibrous tumor/hemangiopericytoma (SFT/HPC) to describe SFT and HPC. Because SFT/HPC has HPC-like features under microscopy and both SFT and HPC show fusion of the NAB2/STAT6.16 genes on chromosome 12q13 (7). HPC can be found anywhere, but the most common locations are the limbs and pelvis. The incidence of HPC in the central nervous system is approximately 1%, but HPC in the spine remains very rare (8, 9), and cases of spinal infiltration are even rarer. Due to the low incidence of spinal cord HPC with spinal cord infiltration, little is known about its clinical characteristics and prognostic factors. Combined with case reports, the clinical data of 11 patients with spinal epidural HPC with spinal cord infiltration were retrospectively analyzed, and univariate statistical analysis was performed to evaluate the prognostic factors that affect RFS.

SFT/HPC, as reported in the literature, is more likely to occur in middle age, with no significant difference between male and female morbidity (10, 11). The average age of our research is 48.82 years, and the male-to-female ratio is 1.75–1; males seemed to be more prone to the disease. The clinical symptoms of HPC are not specific and include neck and back pain, limb movement disorders, sensory disorders, and urinary and stool dysfunction. These symptoms are mostly caused by the mass effect of the tumor, which leads to compression of the spinal cord and nerve roots. MRI scan of the spine is the preferred examination for SFT/HPC but cannot distinguish SFT/HPC from other tumors in the spinal canal (such as meningioma, meningioma, schwannoma). In this group of cases, T2WI showed a slightly higher tumor signal. Therefore, the differential diagnosis of HPC should be considered when the spinal epidural space shows a high signal.

Total surgical resection is the preferred treatment for HPC (12, 13), which is consistent with the conclusion of our analysis. However, massive bleeding during surgery becomes a barrier to total removal. Preoperative endovascular embolization has been shown to be effective in controlling intraoperative bleeding and to facilitate total tumor resection (14). However, when the patient develops severe neurological dysfunction before surgery, vascular embolization may aggravate the patient's symptoms (5). In cases in which the tumor is located in the dura and the mass effect is not obvious, intraoperative bleeding can be reduced by overall resection of the tumor at the interface between the tumor and spinal cord. In our case, the whole tumor was removed with minimal bleeding. A final diagnosis of spinal HPC requires histopathological and immunohistochemical support. Our analysis found that WHO grade III was a predictor of recurrence. This research result is consistent with previously reported (3, 4). A review of the literature found that tumor cells proliferated under a light microscope, some of the nuclei showed atypia, abundant blood vessels were located around the tumor cells, and some of the tumor cells showed typical “staghorn” blood vessels. Immunohistochemistry showed vimentin positivity in all tumors and CD34 positivity in most tumor cells (3, 4). The effect of postoperative radiotherapy remains uncertain, and postoperative radiotherapy had no statistically significant effect on the survival rate of patients in this group. In recent years, stereotactic radiotherapy has become an effective way to treat spinal cord HPC, and the tumor control rate of patients who receive this treatment has been shown to be more than 82% (15–17). Radiosurgery has shown promising application prospects for tumors that cannot be completely resected or recur. Among this group were five patients with recurrence, and the average recurrence time was 23 months. Therefore, a long postoperative follow-up is necessary.

Patients with intraspinal hemangiopericytoma may be more likely to recur than intracranial HPCs. The 5 years RFS rate of intracranial HPC is 85% to 93% (15, 18). It has been reported in the literature that the 5-years RFS of HPC in the spinal canal is 76% (4) and the average RFS is 45.1 months (3). Tumor recurrence is significantly correlated with WHO level III (3, 4). In addition, our findings indicate that the range of tumor resection also affects tumor recurrence. We must point out the biases that may be caused by relatively small sample size limitations and confounding factors. A larger sample size study of multiple factors is needed to further confirm our conclusions.

## Limitations

As a retrospective study, this study has several inherent limitations and should thus be interpreted with caution. Publication bias is unavoidable because unpublished research and studies published in non-English languages were omitted. In addition, some important factors, such as preoperative symptom duration, immunohistochemical factors (such as the Ki-67 index), WHO tumor grade, and tumor size, were not fully reported in all studies and not included in our analysis.

## Conclusion

We report a rare case of spinal intradural HPC with spinal cord invasion in which the tumor was excised en masse under a microscope. Retrospective analysis of the literature found that sex, age, tumor location, and postoperative radiotherapy had no significant impact on the survival rate of patients, and total tumor resection was beneficial to patient survival. The postoperative tumor recurrence time was long, necessitating long-term postoperative follow-up.

## Data Availability Statement

The datasets used and analyzed during the current study are available from the corresponding author on reasonable request.

## Author Contributions

LC, WY, and ZX diagnosed the patient. LC and ZH analyzed literature data. SM analyzed the pathological features of the patients. All authors wrote and revised the manuscript.

## Conflict of Interest

The authors declare that the research was conducted in the absence of any commercial or financial relationships that could be construed as a potential conflict of interest.
